# Patient-Centered Mobile Health Data Management Solution for the German Health Care System (The DataBox Project)

**DOI:** 10.2196/10160

**Published:** 2018-05-11

**Authors:** Titus Josef Brinker, Stefanie Rudolph, Daniela Richter, Christof von Kalle

**Affiliations:** ^1^ Department of Translational Oncology National Center for Tumor Diseases German Cancer Research Center Heidelberg Germany; ^2^ German Cancer Consortium (DKTK) Heidelberg Germany; ^3^ University Hospital Heidelberg Department of Dermatology University of Heidelberg Heidelberg Germany

**Keywords:** medical informatics, health data management

## Abstract

This article describes the DataBox project which offers a perspective of a new health data management solution in Germany. DataBox was initially conceptualized as a repository of individual lung cancer patient data (structured and unstructured). The patient is the owner of the data and is able to share his or her data with different stakeholders. Data is transferred, displayed, and stored online, but not archived. In the long run, the project aims at replacing the conventional method of paper- and storage-device-based handling of data for all patients in Germany, leading to better organization and availability of data which reduces duplicate diagnostic procedures, treatment errors, and enables the training as well as usage of artificial intelligence algorithms on large datasets.

The development of intelligent storage, sharing, and analysis solutions for health care data has evolved over the recent years [1-4]. DataBox is a research project based in Germany and funded by the federal ministry for health as well as the federal ministry for education and research. It aims at improving health data management for patients and health care providers by creating a platform that is accessible from landline phones, computers, mobile phones, and tablets. DataBox provides individual data spaces for storage, analysis, and sharing of health data. The collaborating partners are the National Center for Tumor Diseases in Heidelberg (project lead), Köln University Hospital, Charité University Hospital in Berlin, and the German technology companies SAP and Siemens Healthineers. The ethics committees of the three collaborating centers approved the project.

Currently, patients in Germany receive a printed report by their physician at the end of their stay, often accompanied with other sheets of paper, compact disks, or other physical storage devices containing diagnostic data such as radiological files. Patients are expected to manually carry all this information with them when switching health care providers. This status quo often leads to loss of data, duplicate diagnostic procedures, and treatment errors as well as a lack of instant access to available health data for patients not only during a hospital stay, but also in acute care situations. This lack of data is not only caused by patients losing some of these printed reports, disks, or storage devices or not bringing them to their new care provider, but also due to incompatibility of provided file types between health care providers.

DataBox aims at solving these problems by providing individual data spaces which are accessible for patients of all levels of digital literacy (from landline phones to smartphone devices). Patients can instantly access their individual health data as soon as it is available and share it with selected health care providers of their choice. At the same time, health providers can use the platform to upload health data and to open shared patient data with an integrated format-agnostic viewer.

The digital format of the data enables the training as well as usage of artificial intelligence algorithms on large datasets, ultimately increasing the understandability and value of digitalized health care data for the patient. Machine learning, and more specifically, deep learning algorithms for supervised and unsupervised data analysis, are on the rise in the medical field [5-20] and may be enhanced in their precision by large organized datasets.

The need to give citizens back the control of their data is the current task for health care according to the General Data Protection Regulation [21]. DataBox not only improves access by instantaneously synchronizing the health data in a secured cloud with individual data spaces but also lets the patient choose who may access it.

In the first 18 months (starting in January 2018), the DataBox project will focus on 4,000 lung cancer patients from Germany. However, the vision of the initiators of this government funded project is to replace the status quo as outlined above for the whole German health care system after the 18-month test period.
